# Socioeconomic Factors and Oral Health-Related Behaviours Associated with Dental Caries in Preschool Children from Central Italy (Province of Ascoli Piceno)

**DOI:** 10.1155/2019/7981687

**Published:** 2019-12-23

**Authors:** Alessandro Nota, Silvia Caruso, Tiziana Cantile, Roberto Gatto, Aniello Ingenito, Simona Tecco, Gianmaria F. Ferrazzano

**Affiliations:** ^1^MeSVA, University of L'Aquila, Piazzale Salvatore Tommasi, 1, 67100 Coppito AQ., L'Aquila, Italy; ^2^Dental School, Vita-Salute San Raffaele University, Via Olgettina, 58, 20132 Milan, Italy; ^3^Department of Neuroscience, Reproductive and Oral Sciences, School of Pediatric Dentistry, University of Naples, Federico II, Via S. Pansini, 5, 80100 Naples, Italy; ^4^Unesco Chair in “Health Education and Sustainable Development: Oral Health in Paediatric Age”, University of Naples, Federico II, Naples, Italy

## Abstract

**Background:**

Dental caries is a serious oral health concern with high prevalence in preschool children.

**Aim:**

To assess the association among oral health-related behaviors, socioeconomic factors, and dental caries in Italian preschool children.

**Design:**

513 preschool children from 3 to 6 years of age, enrolled in nursery schools in central Italy, represent the population of the present study. The children underwent dental clinical examination and were divided on the basis of their caries experience in the primary dentition (“Yes” or “No”). Nonparametric analysis and univariate and multivariate logistic regression models were applied to evaluate the contribution of oral health-related behaviors and socioeconomic factors to the caries experience in the primary dentition.

**Results:**

419 children were caries free, and 94 children had caries experience in the primary dentition, corresponding to the 18.4% of participants. Different oral health-related behaviors and socioeconomic factors resulted to be associated with caries development. In particular, the mother's foreign nationality results in a strong predictive factor.

**Conclusions:**

The multivariate logistic model revealed factors significantly affecting caries experience in the primary dentition, which were the mother's nationality, the number of dental visits in a year, and the personal impression by the mother.

## 1. Introduction

Although over the last decades, epidemiological data reveal a significant decline in caries prevalence in children and adolescents from industrialized Western countries [1, 2], dental caries is still a common oral disease, with a prevalence ranging around 60–90% [3, 4], even diffused among preschool children. Dental caries negatively affects children's quality of life by their psychological and social relations, also causing feelings of discomfort from an early age [5, 6]. Furthermore, dental caries leads to considerable cost in the short and long term [7].

For these reasons, a reduction in the prevalence of dental caries is desirable [8] and identifying high-risk populations for developing dental caries can help in achieving this goal by targeting the right audience with preventive strategies [7, 9].

Several studies and a recent meta-analysis have shown that education level and socioeconomic status (SES) of children's parents significantly influence their oral health status [10–12]. This could probably be related with the strong influence of these factors on the dietary habits [13, 14], the consumption of sweets being an important cause in the aetiology of caries, together with teeth form, salivary flow [15], and oral hygiene [16, 17]. There is a convincing evidence of an association between the amount and frequency of sugary snacks and an increased risk of caries [18], but different predictors could play a role in determining the final influence of the family's SES on children's dental health. Additionally, the influence of SES on dental health has been shown to be stronger for preschool children than older children [19, 20], because the preschool period is the time when wrong oral health habits, caries patterns, and risk factors are being established, and thus, it is the ideal time to act with preventive strategies to establish healthy trends which can have a lifelong influence [21, 22].

Thus, identifying the risk predictors in preschool children can be useful to set a primary prevention in this population and maintain a low caries risk status [23].

In the literature, there are few studies based on European samples assessing that SES significantly influence the development of dental caries in preschool children [3, 10, 19].

However, in Italy, this kind of studies did not involve the entire national territory, but only samples from Italian islands, reflecting the conditions of a particular population [2, 20].

They stated an overall prevalence of dental caries of 38.56% among 5-year olds [2] and of 42.5–57.5% among 6-year olds [20], and the maternal educational level was found to be a protective factor for the presence of caries [2] and also associated with children's caries severity [20].

Furthermore, this aspect was insufficiently analysed in Southern Europe, limiting the development of preventive strategies in this area.

Thus, the aim of this cross-sectional study was to assess the association among oral health-related behaviors, socioeconomic factors, and dental caries in Italian preschool children.

## 2. Materials and Methods

The present survey was performed in 10 public schools belonging to “Unione dei Comuni Vallata del Tronto,” in the province of Ascoli Piceno (Marche, Italy) in central Italy. The criteria of selection for the 10 schools were them belonging to the same region of central Italy, the availability of the school director to carry out the project, and the possibility of conducting clinical examinations observing the appropriate health rules.

The project consisted of a clinical examination in a classroom and the distribution of questionnaires to be completed by children's parents at home.

Parents were preliminary informed about the aims and methodologies and encouraged to submit their children to the project. They were also asked to sign a proper informed consent form, after which their children were enrolled in the study.

A total sample of 514 preschool children from 3 to 6 years (280 males and 233 females; mean age 4.58 years) were enrolled in the study, all attending the 1st, the 2nd, or the 3rd year of nursery school, among which 484 children were native Italians, while 30 children were from other ethnic groups.

The survey lasted complessively eight days.

First, the children underwent the clinical examination in comfortable classrooms, with good lighting. Disposable kits, consisting of disposable gloves, a dental probe, a mouth mirror, and a pair of tweezers, were used.

Dental caries diagnosis was assessed according to the visual examination method by the British Association for the Study of Community Dentistry (BASCD): all surfaces of each eligible tooth were examined and scored. Caries was diagnosed visually at the “caries into dentine” level. In cases of doubt, the visual examination was followed, if needed, by a dental probing (by using a dental explorer *no*. 23). [24] At the end of the clinical examination, each child was categorized on the basis of his/her caries experience in the primary dentition (“Yes” when dmf-t was >0 and “No” for caries-free children).

The clinical examinations were conducted by one calibrated examiner: the examiner calibration has been previously realized over 30 children (equivalent to the 36.5% of the whole sample to examine), recruited in the Paediatric Dentistry Department of the University of L'Aquila (Italy) where the entire survey was projected. The results over 30 preschool children were that there was a 100% agreement between the diagnoses made by the operator and those made by 2 expert colleagues after a customary dental examination on the professional chair and with appropriate light, with full sensitivity and specificity.

In the second phase of the project, after the clinical examination in the classrooms, a questionnaire about mouth hygiene, eating habits, and socioeconomic factors were given to the parents of the involved children asking them to complete it honestly and appropriately, at home.

Data of the questionnaire were recorded and reported in a descriptive analysis in order to illustrate the characteristics of the two groups: children with caries experience in the primary dentition, *versus* caries-free children.

Frequency and percentages were calculated for discrete and nominal variables, while quantitative variables were evaluated through the median and the interquartile range (IQR). Since the data have a nonnormal distribution according to the Shapiro–Wilk test, a nonparametric analysis was conducted in order to determine the differences between the average values, and the *χ*^2^ test or Fisher's exact test was applied to evaluate the frequency differences between the two groups.

Univariate and multivariate logistic regression models were used. In these models, the caries experience (“Yes” or “No”) was considered as a dependent variable, and the contribution to this condition of demographic, socioeconomic, eating and oral hygiene habits was considered as explanatory variables.

In order to study the relationship between the dependent variable and the explanatory variables, a cross-tabulation analysis was initially performed among groups and the *χ*^2^ test was used. Then, only the variables with a statistically significant association with caries experience were introduced to the logistic regression model, with the presence/absence (“Yes” or “No”) of caries experience as dependent variable. For each analysis, the threshold for statistical significance was set at *p* < 0.05.

The data were processed through the statistical package STATA/IC 12.0.

The study was conducted in accordance with the Declaration of Helsinki (1964) and approved by the ethical committee of the University of L'Aquila, Italy.

## 3. Results

Out of the whole sample of 514 children, 419 participants (228 males, 191 females) were caries free, and 95 children had caries experience in the primary dentition (53 males, 42 females) corresponding to the 18.4% of participants.

The *χ*^2^ test assessed that age and gender were not significantly associated with the dependent variable (respectively, *p*=0.109 and *p*=0.874). The most affected teeth were the second lower primary molars, followed by the first lower primary molars and by the first upper primary molars.

The characteristics of the whole sample were as follows: the majority of children had Italian parents with high school level of education and came from families with an average income between €12.000 and €24.000 and home ownership (Table 1).

Regarding the eating habits, the great part of the sample reported to have 2-3 meals a day, without eating sugars before going to bed (Table 2). The majority of the sample had received breastfeeding for a period of about 10 months (Table 2).

Regarding oral health, the great part of the sample reported brushing teeth about 2 times a day, even when tired; they have not been visited by a dentist before the present project, and they have never received any fluoride application on teeth, although they used to brush his/her teeth with a fluoride toothpaste (Table 2).

In addition, the majority of children has not received teeth sealings yet (Table 2).

Table 3 shows the results of univariate and multivariate logistic regression models.

The unadjusted analysis evidenced a number of factors that, taken individually, can affect the caries experience in the primary dentition, which are the mother's non-Italian nationality (*p*=0.001); a family income lower than 12000 Euro a year (*p*=0.002); the absence of a family own residence (*p*=0.003); a number of 2 times that the patient habitually eats between the two meals (*p*=0.002), a number of dental visits in a year (*p*=0.004); the consumption of candies and sweets between meals (*p*=0.003); the consumption of sweet drinks or sweet food before going to bed (*p*=0.042). Finally, the mothers' perception of children's caries experience was also significantly associated with the caries experience (*p*=0.001).

The adjusted multivariate step revealed that factors significantly affecting the caries experience were the mother's non-Italian nationality; the number of dental visits in a year; and concerns of caries expressed by the mother.

Mothers with non-Italian nationality were 6.24 times (C.I. 11.12–18.22; *p*=*p*.004) more exposed to caries experience of their children, compared to Italian mothers. And a personal impression of the mothers about the caries experience of their children was also a significant predictor. In addition, patients who received almost one visit for year were less exposed to caries experience compared to children who did not.

## 4. Discussion

The present study analysed a sample of preschool children in central Italy, demonstrating that some socioeconomic factors and habits resulted as risk factors for dental caries development in preschool children. Among the socioeconomic factors, the maternal foreign nationality was identified as the main risk factor.

A similar result was also reported in northern Europe (the Netherlands) by van der Tas et al. [3, 7] that observed a similar result in a greater sample than the present one.

Those authors stated that the maternal education level and their foreign nationality are the most influential risk factors for caries development in preschool children.

Thus, both in northern and southern Europe, differently from other continents, there seems to exist a negative association between the immigrant status and oral health, as also reported by Noymer and Lee [25] for the general health.

In Italy, Carta et al. [20] and Pizzo et al. [2], respectively, from Sardinia and Sicily islands, observed a strongest negative influence of the maternal education level on their preschool children's oral health, rather than economic factors, data which agree with the present study.

Educational level is an important marker of socioeconomic condition and maternal educational level was previously identified as one of the best predictors in all countries for children's health including oral health [2, 10, 26], especially when the disease prevalence is high [13].

Previous studies enlightened a gap between Italy and other EU countries, where there is a screening program across the population (infantile, adolescent, and adult) at regular intervals of ten years, which allowed, from 1973, to reduce drastically the prevalence of caries, especially in preschool age [27–29]. On the contrary, the preschool caries risk pattern in Italy appears to be similar to that reported by van der Tas et al. [3] and the caries-free participants percentage even higher in Italy (81.6% vs 77.1%). This is still far from what was asked by the World Health Organization (WHO) targets for 2010, which were to provide for a reduction of caries, with an average of caries-free participants equal to approximately 90% among preschool children [30].

This difference from the reported gap may be due to the variation of several heterogeneous factors, which could be represented by disparities in socioeconomic development, dietary habits, parental attention and attitudes in child oral hygiene, and access to public dental health service [31, 32].

The last aspect, as demonstrated in the international scientific literature, can play an important role in influencing caries distribution: the presence of public dental care services and of national preventive programs can contribute to reduce caries in populations with least social, economical, and cultural levels and can also balance the disparities between local and immigrant people [33].

In particular, analysing the dietary habits and parental attention to children's dental health, the present study showed a predominance of the reduced assiduousness to the dental specialist as a primary caries risk factor in preschool children.

Central Italy, analysed in this study, is particularly representative of the whole nation being observed as an intermediate situation between the northern and southern parts of the country. A marked discrepancy between the various ethnic groups, with a notable presence of caries in children of foreign origin, was also previously observed [33] and confirmed by the findings of the present study. This study showed the necessity to carry out more epidemiological surveys fairly distributed across the national territory and consequently to implement an ever-increasing number of prevention campaigns among the populations that may involve dental practitioners, paediatricians, teachers, parents, and children in order to reduce the incidence of the disease.

Disparities in socioeconomic development, dietary habits, parental attention and attitudes in child oral hygiene, and access to public dental health services represent important risk factors for the development of dental caries in preschool children. In particular, the maternal foreign origin seems to be related to a higher caries risk among other factors. Data analysis suggests monitoring of the preschool children population over time through future preventive surveys and planning local preventive actions, aimed to increase the caries-free participants percentage in the local population considering socioeconomic and daily habits predictive factors, especially in relationship with the increasing immigration in southern Europe during the last decades. The data obtained from the examined sample are acceptably in line with what were observed in northern Europe.

On the basis of the present results, an appropriate strategy to reduce the discrepancy in the oral health of children consists in implementing the educational programs aimed to instruct their mothers (in particular those with foreign nationality) in order to raise their attention to their children's oral health. These information and prevention programs could be implemented in kindergartens, which are a point of socialization and integration of families. This would further improve the educational offer of the schools that carry them out.

This study was limited because the sample was from a single region of Central Italy; thus, the results cannot be generalized to the whole population from southern Europe.

In addition, another limit of the present investigation is that the data evidences caries experience, but caries severity (number of decayed, filled, or missed teeth) was not evaluated.

## 5. Conclusions

Out of the whole sample of 514 preschool children from Central Italy (the province of Ascoli Piceno) (aged from 3 to 6 years; mean age: 4.58 years), 419 participants (228 males, 191 females) were caries free, and 95 children showed caries experience in the primary dentition (53 males, 42 females), corresponding to the 18.4% of participants. The multivariate logistic model revealed that factors significantly affecting the presence of caries were the mother's nationality, the number of dental visits in a year, and caries concerns expressed by the mother.

## Figures and Tables

**Table 1:** Socioeconomic factors associated with caries experience in the primary dentition (*N* = 514).

Socioeconomic factors	*N*	Caries experience in the primary dentition		*p* value
		“No” *n* (%)	“Yes” *n* (%)	
Mother's nationality				
Italian, *n* (%)	259	228 (88.0%)	31 (11.0%)	0.001^*∗∗*^
Non-Italian, *n* (%)	30	13 (43.3%)	17 (56.7%)	

Father's nationality				
Italian, *n* (%)	264	230 (87.2%)	34 (12.8%)	0.001^*∗∗*^
Non-Italian, *n* (%)	24	10 (41.7%)	14 (58.3%)	

Income				
Higher than €36.000, *n* (%)	39	35 (55.5%)	4 (44.5%)	0.001^*∗∗*^
Between €24.000 and €36.000, *n* (%)	80	70 (84.0%)	10 (16.0%)	
Between €12.000 and €24.000, *n* (%)	106	89 (87.5%)	17 (12.5%)	
Lower than €12.000, *n* (%)	36	20 (89.7%)	16 (10.3%)	

Own residence				
Yes, *n* (%)	221	192 (86.8%)	29 (13.2%)	0.002^*∗∗*^
No, *n* (%)	65	46 (70.8%)	19 (29.2%)	

Mother's title of study				
University degree, *n* (%)	48	42 (87.5%)	6 (12.5%)	0.028^*∗*^
High school diploma, *n* (%)	158	137 (86.7%)	21 (13.3%)	
Middle and primary school, *n* (%)	80	59 (73.7%)	21 (26.3%)	

Father's title of study				
University degree, *n* (%)	27	25 (92.6%)	2 (7.4%)	0.157 ns
High school diploma, *n* (%)	152	129 (84.9%)	23 (15.1%)	
Middle and primary school, *n* (%)	107	84 (78.5%)	23 (21.5%)	

^*∗∗*^ *p* < 0.01 (*χ*^2^ test or Fisher's exact test); ^*∗*^*p* < 0.05 (*χ*^2^ test or Fisher's exact test); ns: not significant.

**Table 2:** Association among caries experience in the primary dentition and the answers to the questionnaire concerning oral health-related behaviors.

Questions	*N* (*N* = 513)	Caries experience in the primary dentition		*p* value
		“No” (*n* = 419) median (IQR)	“Yes” (*n* = 94) median (IQR)	
How often does the patient brush his teeth?	2 (1-2)	2 (1-2)	2 (1-2)	^0.077 ns

How often does the patient eat between meals?				
Median (IQR)	1 (1-2)	1 (1-2)	2 (1-3)	^0.002^*∗∗*^

Does the patient often have candies and sweets between meals?				
No	147	132 (89.8%)	15 (10.2%)	°0.003^*∗∗*^
Yes	141	108 (76.6%)	33 (23.4%)	
If yes, how often?				^0.671 ns
Median (IQR)	1 (1-2)	1 (1-2)	1 (1-2)	

Has the patient ever been visited by a dentist?				
Yes	105	83 (79.0%)	22 (21.0%)	°0.139 ns
No	183	157 (85.8%)	26 (14.2%)	
If yes, how many times a year?				^0.001^*∗∗*^
Median (IQR)	1 (1-2)	1 (1-2)	1 (1-2)	

Topical fluoride application?				
Yes	47	39 (82.98)	8 (17.02)	°0.924 ns
No	237	198 (83.54)	39 (16.46)	

Has the patient taken or is he still taking fluoride?				
Yes	116	100 (86.21)	16 (13.79)	°0.273 ns
No	171	139 (81.29)	32 (18.71)	

Is the patient using a fluoride toothpaste?				
Yes	228	189 (82.89)	39 (17.11)	°0.734 ns
No	59	50 (84.75)	9 (15.25)	

Does the patient have sealings?				
Yes	11	7 (63.64)	4 (36.36)	°0.075 ns
No	276	232 (84.06)	44 (15.94)	

Breastfeeding?				
Yes	242	199 (82.23)	43 (17.77)	°0.139 ns
No	45	41 (91.11)	4 (8.89)	
If yes, how long?				
<10 months	161	137 (85.09)	24 (14.91)	°0.101 ns
≥10 months	81	62 (76.54)	19 (23.46)	

Do you think your child may have caries?				
No	244	223 (91.39)	21 (8.61)	°0.001^*∗∗*^
Yes	43	17 (39.53)	26 (60.47)	

Does the patient have sweet drinks or sweet food before going to bed?				
No	201	174 (86.57)	27 (13.43)	°0.039*∗*
Yes	86	66 (76.74)	20 (23.26)	

When (age in months) did you start brushing his/her teeth?				
Median (IQR)	24 (15 36)	24 (12 36)	29 (24 36)	^0.010^*∗∗*^

Do you always brush your child's teeth even when tired?				
Always	80	66 (82.50)	14 (17.50)	°0.891 ns
Often	111	94 (84.68)	17 (15.32)	
Sometimes	83	70 (84.34)	13 (15.66)	
Never	13	10 (76.92)	3 (23.08)	

Do you think children's teeth are important?				
Of course	225	188 (83.56)	37 (16.44)	°0.989 ns
Yes	55	46 (83.64)	9 (16.36)	
No	7	6 (85.71)	1 (14.29)	

°*χ*^2^ test or Fisher's exact test; ^Mann–Whitney test; ^*∗∗*^*p* < 0.01; ^*∗*^*p* < 0.05; ns: not significant.

**Table 3:** Results from the logistic regression analysis considering the caries experience as dependent variable.

Variable	Association between caries experience in primary dentition and the considered variables using a univariate logistic regression model.			Multivariate logistic regression model		
	OR	95% CI (confidence interval)	*p* value	OR	95% CI (confidence interval)	*p* value
Mother's nationality						
Italian°	1			1		0.004^*∗∗*^
Non-Italian	8.64	4.11–22.9	0.001^*∗∗*^	6.24	1.12–18.22	

Income						
Higher than €36.000°	1			1		
Between €24.000 and €36.000	1.25	0.37–4.27	0.722 ns	1.32	0.37–4.72	0.668 ns
Between €12.000 and €24.000	1.67	0.53–5.32	0.384 ns	1.23	0.37–4.15	0.735 ns
Lower than €12.000	7	2.05–23.85	0.002^*∗∗*^	3.56	0.88–14.45	0.075 ns

Own residence						
Yes°	1			1		
No	2.73	1.41–5.30	0.003^*∗∗*^	1.71	0.74–3.98	0.211 ns

Number of times the patient eats between the meals	1.74	1.23–2.47	0.002^*∗∗*^	0.49	0.21–1.18	0.112 ns

Candies and sweets between meals:						
No°	1	1.39–5.21	0.003^*∗∗*^	1	1.01–3.22	0.124 ns
Yes	2.69			1.32		

Number of dental visits every year	2.36	1.32–4.20	0.004^*∗∗*^	3.43	1.18–9.95	0.024^*∗*^

Do you think your child may have caries?						
No°	1			1		
Yes	16.24	7.61–34.64	0.001^*∗∗*^	6.59	1.19–36.48	0.031^*∗*^

Does the patient have sweet drinks or sweet food before going to bed?						
No°	1			1		
Yes	1.95	1.03–3.72	0.042^*∗*^	3.54	0.01–45.92	0.143 ns

^*∗∗*^ *p* < 0.01; ^*∗*^*p* < 0.05.

## Data Availability

The data used to support the findings of this study are included within the article.
